# Threats to public health in Peru and the response of the Instituto Nacional de Salud

**DOI:** 10.17843/rpmesp.2022.392.11906

**Published:** 2022-06-30

**Authors:** Víctor Suarez, César Cabezas

**Affiliations:** 1 Instituto Nacional de Salud, Lima, Peru. Instituto Nacional de Salud Lima Peru

Two and a half years have elapsed since the beginning of the COVID-19 pandemic, with 547,549,338 cases reported to date, 6,336,074 deaths, and 11,745,435,307 doses administered worldwide^ (^ ^1^. In Peru, 3,616,929 cases have been reported with 213,462 deaths, and 77,808,132 doses of vaccine have been administered ^2^. No country has remained unaffected, although the impact in developed countries has been different from that in developing countries, everywhere the pandemic has exposed the various limitations of health systems. This has been reflected both in the collective health component and in the provision of services and, in particular, has revealed the serious inequities and the social and economic determinants that exist in Latin American countries. A similar picture is emerging with a new public health threat that reminds us that many of these elements remain unimproved, the current outbreak of simian smallpox or monkeypox; which, similar to COVID-19 ^3^, has started in European countries and is currently spreading to southern countries, which in the long term may end up with higher morbidity rates than where the epidemic began.

In this context, since the beginning of the pandemic, the National Institute of Health (INS) of Peru, has played an active role in the control of these health emergencies through its national centers and within the scope of its competence. Probably the most outstanding example is the strengthening of molecular diagnostics in the country through the consolidation of the National Reference Laboratory and the support to the National Network of Public Health Laboratories. Thus, at the beginning of the pandemic there was only one central laboratory for the molecular diagnosis of SARS-CoV-2 with the Reverse Transcription Polymerase Chain Reaction (RT-PCR) technique; currently there are 127 laboratories performing this test (five laboratories of the INS; 28 of the integrated health network directorates and regional health directorates; 13 of the Ministry of Health; one of the Armed Forces; 12 of EsSalud; six of universities and 62 of private laboratories). In this way, diagnosis has been decentralized and early detection and epidemiological surveillance of this infection has been strengthened in many regions of the country. Moreover, to reach remote areas, three mobile laboratory units called “COVID Maskaq” were implemented, which travel to different areas of the country that do not yet have molecular technology. Innovative technologies were also used, such as isothermal molecular tests (LAMP), which have less complex laboratory requirements and can be implemented at the first level of care. It is in this network of laboratories, initially established for COVID-19, that the implementation of RT-PCR for simian smallpox virus and any technique required in the future for molecular diagnosis of emerging and endemic infectious diseases is envisioned.

When SARS-CoV-2 variants appeared in the world, it was necessary to implement whole genome sequencing technique to this pandemic virus, the technique was performed at INS for other etiologies and was implemented not only at INS, but also in three regions (Piura, Junin and Cusco). To date, 20,961 complete SARS-CoV-2 genome sequences have been produced from samples from all over the country, which places Peru in third place in South America, after Brazil and Chile. This has allowed us to identify, during the different pandemic waves, the lambda, delta, and omicron variants as well as their descendant lineages such as BA2, BA2.12.1, BA.4 and BA.5; this information is registered and freely available in the GISAID database (https://www.gisaid.org/), which allows us to actively contribute to the surveillance of the pandemic in the country and to guide prevention and control strategies, before and after the immunization process. The regular implementation of this technique for SARS-CoV-2 has allowed us to adapt it, in a short time, to the simian smallpox virus, identifying, through the sequencing of the first cases, at least three different phylogenetic groupings, which indicate at least three parallel introductions of the virus in Peru. Further genomic sequencing of the simian smallpox virus will allow us to better understand its transmission dynamics and make better decisions for its control.

The promotion of research on COVID-19 has boosted the study and technological development of the molecular diagnosis of SARS-CoV-2, viral isolation, ELISA techniques to detect antibodies, and the evaluation of the effectiveness of vaccines against COVID-19. Although, compared to other countries, research funding has been relatively modest, several of the generated tools have been implemented under routine conditions and could be adapted to other emerging diseases, such as the LAMP technique for point-of-contact testing.

In addition to specialized diagnosis and laboratory surveillance, which are the most widely known tasks of the INS, several areas have deployed control actions towards the health emergency caused by COVID-19 and are currently supporting the control of monkeypox, being prepared to face future health challenges. Thus, the Unit for Analysis and Generation of Evidence in Public Health (UNAGESP) has generated evidence on several aspects of the pandemic, such as prevention (vaccines) and pharmacological and non-pharmacological strategies for pandemic control, in order to provide necessary information for decision making regarding the control of COVID-19. Regarding the occupational health component, the National Center for Occupational Health and Environmental Protection for Health (CENSOPAS) has been carrying out since 2020 the registration of protocols for the surveillance of COVID-19 in workplaces, and document inspection in coordination with the National Superintendence of Labor Inspection (SUNAFIL) of the Ministry of Labor. On the other hand, the National Quality Control Center (CNCC) carries out the control of products related to the prevention and control of COVID-19, such as the filtration and fit of masks and medical oxygen plants. The National Center for Food and Nutrition (CENAN) promotes healthy eating as a relevant element for the reduction of vulnerability to COVID-19 with balanced and low-cost recipes for the people who go to the “common pots” in Lima and in different regions of the country.

Scientific dissemination during the pandemic was carried out by the *Revista Peruana de Medicina Experimental y Salud Pública*, which fulfills the dissemination role and highlights contributions from Peruvian authors researching aspects of SARS-CoV-2 and COVID-19. In the second issue of 2020, 15 articles on COVID-19 were published; in the third issue, 11 were published, and in the fourth, an additional 11. In 2021, a total of 103 articles on COVID-19 were published; there are 556,693 visits and 1,007,246 downloads of articles from the journal’s web page. The journals’ website has 556,693 visits and 1,007,246 downloads of full-text articles. In this way we contributed with scientific evidence, in order to inform readers and the general population.

However, it should be emphasized that the contributions of the INS to the control of the COVID-19 pandemic, smallpox and other health emergencies, are nothing more than the fulfillment of the mission entrusted to our institution, whose objective is, precisely, to ensure that science contributes to the country’s health policy, through research and scientific evidence. Despite what was learned during the pandemic, a rigorous analysis of what happened as well as of the lessons learned and recommendations is essential to achieve an adequate national reconstruction of the health system, taking as a premise that health is a human right and a public good. It is necessary to rethink what has been done at different levels, taking into account the successes and failures in order to better prepare ourselves for future events, including global warming and the epidemics it may bring. It is therefore urgent to achieve real access to health and strengthening primary health care, which is extremely necessary during a health emergency, in order to assist the most vulnerable populations with a multisectoral approach that includes social support ^4^.

The new health intelligence approach required to address pandemics and epidemics should include modern approaches to surveillance and risk assessment, as well as greater trust and cooperation among stakeholders and society, including academia and the media ^5^. Traditional surveillance approaches, such as recording the number of cases and deaths, are insufficient for upcoming health threats. Another key element will be the cooperation among countries, especially among South American countries, to better face together and with modern consensual approaches the coming challenges, adapting to changes and re-evaluating our policies in real time, which is what the control of current infectious diseases demands.

From the INS, there is Decree Law 1504 (approved in 2020) which aims to “Strengthen the National Health System to ensure intra and inter-sectoral, intergovernmental, public and private entities, and the population for the fulfillment of health policies; strengthening the health sector, through the organization and efficient management of the Instituto Nacional de Salud in favor of public health in the country; improving the surveillance and prevention of diseases, outbreaks, endemics, epidemics and pandemics in the national territory; contributing to the control of diseases that affect the lives of the population, as well as improving research, development and innovation, and health technologies with the participation of public and private institutions and the academic sector”.

To this end, and among other initiatives, the investment project for the “Improvement and Expansion of the Services provided by the National Public Health Surveillance System”, co-financed by the World Bank, is currently underway. This project aims to strengthen the national response capacity to emerging and reemerging diseases that affect the health of the population, strengthening both the INS and the 24 regions and three macro-regional centers in the North, South and East, which will be able to achieve an effective decentralization of the specialized services provided by the institution.

Finally, considering that during the 1970s the INS contributed to the eradication of smallpox in Peru and other South American countries through the production of freeze-dried smallpox vaccine, legislative initiatives have been proposed and supported in order to have a vaccine production plant with modern technological platforms, and thus provide the country with technical capabilities for a timely response against epidemics and endemics of neglected diseases, in addition to pandemics. “May the next pandemic find us better prepared as Peruvians and as human beings”.
