# Supplementary material for: Threats to public health in Peru and the response of the Instituto Nacional de Salud
Source: Rev Peru Med Exp Salud Publica. 2022 Jun 30;39(2):140–2. doi: 10.17843/rpmesp.2022.392.11906 (PMC11397592; doi:10.17843/rpmesp.2022.392.11906)
Supplement: Translation

# Translation: Amenazas a la salud pública en el Perú y la respuesta del Instituto Nacional de Salud

**Authors:** Víctor Suarez, César Cabezas

**Affiliations:** 1 Instituto Nacional de Salud, Lima, Perú.

Han transcurrido dos años y medio desde el inicio de la pandemia de la COVID-19, habiéndose reportado a la fecha 547 549 338 casos, 6 336 074 defunciones en el mundo, y 11 745 435 307 dosis de vacunas administradas ^1^. En el Perú se han registrado 3 616 929 casos con 213 462 defunciones y se han aplicado 77 808 132 dosis de vacunas ^2^. Ningún país ha dejado de ser afectado, si bien el impacto en los países desarrollados ha sido distinto a los países en desarrollo, en todas partes la pandemia ha visibilizado las diversas limitaciones de los sistemas sanitarios. Esto se ha reflejado tanto en el componente de salud colectiva, como en la atención de servicios y, en particular, se han desnudado las serias inequidades y los determinantes sociales y económicos que existen en los países latinoamericanos. Un panorama similar se vislumbra con una nueva amenaza a la salud pública que nos recuerda que muchos de estos elementos siguen sin mejorar, el actual brote de viruela símica; de manera similar a la COVID-19 ^3^ se ha iniciado en los países europeos y en la actualidad se está extendiendo a los países del sur, quienes a largo plazo pueden terminar con mayores tasas de morbilidad que donde comenzó la epidemia. 

En este contexto, el Instituto Nacional de Salud (INS) de Perú, desde el inicio de la pandemia, ha tenido un rol activo en el control de estas emergencias sanitarias a través de sus centros nacionales y en el ámbito de su competencia. Probablemente, el ejemplo más resaltante es el fortalecimiento del diagnóstico molecular en el país a través de la consolidación del Laboratorio de Referencia Nacional y el apoyo a la Red Nacional de Laboratorios de Salud Pública. Es así que al inicio de la pandemia se contaba solamente con un laboratorio central para el diagnóstico molecular del SARS-CoV-2 con la técnica de Reverse Transcription Polymerase Chain Reaction (RT-PCR), actualmente se tienen 127 laboratorios que realizan esta labor (cinco laboratorios del INS; 28 de las direcciones de redes integradas de salud y de las direcciones regionales de salud; 13 del Ministerio de Salud; uno de las Fuerzas Armadas; 12 de EsSalud; 6 de universidades y 62 de laboratorios privados). De esta manera, el diagnóstico se ha descentralizado y fortalecido la detección temprana y la vigilancia epidemiológica de esta infección en muchas regiones del país. Es más, para llegar a zonas lejanas se implementaron tres unidades de laboratorios móviles denominados «COVID Maskaq», que se desplazan por diferentes zonas del país que todavía no cuentan con la tecnología molecular. También se comenzaron a utilizar tecnologías innovadoras, como las pruebas moleculares isotermicas (LAMP), las cuales tienen requerimientos de laboratorio menos complejos y pueden ser implementadas en el primer nivel de atención. Es en esta red de laboratorios, afianzada inicialmente para la COVID-19, se vislumbra implementar la RT-PCR para el virus de la viruela símica y cualquier técnica que se requiera en el futuro para diagnóstico molecular de enfermedades infecciosas emergentes y endémicas.

Cuando en el mundo aparecieron las variantes del SARS-CoV-2, fue necesario implementar la técnica de secuenciación de genoma completo a este virus pandémico, la técnica se realizaba en el INS para otras etiologías y se implementó no solo en el INS, sino también en tres regiones (Piura, Junín y Cusco). Hasta la actualidad se han producido 20 961 secuencias completas de genoma del SARS-CoV-2 de muestras provenientes de todo el territorio nacional, lo cual nos sitúa en el tercer lugar de Sudamérica, luego de Brasil y Chile. Esto ha permitido identificar en las distintas olas pandémicas a las variantes lambda, delta, ómicron y sus linajes descendientes como BA2, BA2.12.1, BA.4 y BA.5, información registrada y de libre disposición en la base de datos GISAID (https://www.gisaid.org/) que permite contribuir activamente a la vigilancia de la pandemia en el país y orientar las estrategias de prevención y control, antes y después del proceso de inmunización. Haber implementado de manera regular esta técnica para el SARS-CoV-2 ha permitido adaptarla, en poco tiempo, al virus de la viruela símica, logrando identificar, a través de la secuenciación de los primeros casos, por lo menos tres agrupaciones filogenéticas distintas, las cuales indican, por lo menos, tres introducciones paralelas del virus en el Perú. Seguir realizando el procedimiento de secuenciación genómica al virus de la viruela símica permitirá conocer mejor su dinámica de transmisión y tomar mejores decisiones para su control. 

Respecto a la promoción de la investigación sobre la COVID-19, se ha impulsado la investigación y desarrollo tecnológico en el diagnóstico molecular del SARS-CoV-2, así como el aislamiento viral, desarrollo de técnicas de ELISA para detectar anticuerpos, y evaluación de la efectividad de las vacunas contra la COVID-19. Si bien, en comparación con otros países, el financiamiento para investigación ha sido relativamente discreto, varias de las herramientas generadas han podido ser implementadas en condiciones de rutina y podrán ser adaptadas a otras enfermedades emergentes, como la técnica de LAMP para diagnóstico en el punto de contacto. 

Además del diagnóstico especializado y la vigilancia laboratorial, que son las labores más conocidas del INS, varias áreas han desplegado sus acciones de control hacia la emergencia sanitaria ocasionada por la COVID-19 y, actualmente, vienen apoyando en el control de la viruela del mono, encontrándose preparadas para enfrentar los futuros desafíos sanitarios. Es así como la Unidad de Análisis y Generación de Evidencias en Salud Pública (UNAGESP) ha generado evidencias sobre diversos aspectos de la pandemia, como la prevención (vacunas); estrategias farmacológicas y no farmacológicas para el control de la pandemia; para proporcionar información necesaria en la toma de decisiones respecto al control de la COVID-19. En el componente de salud ocupacional, el Centro Nacional de Salud Ocupacional y Protección del Ambiente para la Salud (CENSOPAS) realiza desde el 2020 el registro de protocolos para la vigilancia de la COVID-19 en los centros de trabajo, y la fiscalización documentaria en coordinación con la Superintendencia Nacional de Fiscalización Laboral (SUNAFIL) del Ministerio de Trabajo. Por otro lado, el Centro Nacional de Control de Calidad (CNCC) realiza el control de productos relacionados con la prevención y control de la COVID-19, como el filtro y ajuste de mascarillas, y las plantas de oxígeno medicinal. El Centro Nacional de Alimentación y Nutrición (CENAN) realiza la promoción de la alimentación saludable, como elemento relevante para la reducción de la vulnerabilidad frente a la COVID-19 con recetarios balanceados y de bajo costo para los pobladores que acuden a las ollas comunes en Lima y en diferentes regiones del país.

La difusión científica durante la pandemia se realizó a través de la *Revista Peruana de Medicina Experimental y Salud Pública* que cumple el papel de difusión y destaca contribuciones de autores peruanos que investigan aspectos del SARS-CoV-2 y de la COVID-19. En el segundo número del 2020 se publicaron 15 artículos sobre la COVID-19; en el tercer número se publicaron 11, y en el cuarto otros 11; en el 2021 se han publicado en total 103 artículos. Actualmente, la página web de la revista registra 556 693 visitas y 1 007 246 descargas de artículos a texto completo. De esta forma se contribuyó con evidencia obtenida mediante el método científico, a fin de que los lectores y la población general se encuentre informada. 

Sin embargo, se debe resaltar que todo el aporte del INS al control de la pandemia por la COVID-19, la viruela símica y otras emergencias sanitarias, no es más que el cumplimiento de la misión encomendada a nuestra institución, cuyo objetivo es, justamente, velar por que la ciencia contribuya a la política sanitaria del país, a través de la investigación y de la evidencia científica. A pesar de lo aprendido durante la pandemia, es imprescindible un proceso riguroso de análisis de lo ocurrido y de generación de lecciones aprendidas y recomendaciones para lograr una adecuada reconstrucción nacional del sistema de salud, teniendo como premisa que la salud es un derecho humano y un bien público. Es necesario repensar lo actuado en los diferentes niveles, recabando los aciertos y desaciertos con el fin de prepararnos mejor para próximos eventos, incluyendo el calentamiento global y las epidemias que traiga consigo. Por ello, es urgente lograr el acceso real a la salud, fortaleciendo la atención primaria, sumamente necesaria durante la emergencia sanitaria, a fin de asistir a las poblaciones más vulnerables con un enfoque multisectorial que incluya el apoyo social ^4^. 

El nuevo enfoque de inteligencia sanitaria que se requiere para afrontar pandemias y epidemias deberá incluir enfoques modernos de vigilancia y evaluación de riesgos, así como una mayor confianza y cooperación entre las partes interesadas y la sociedad, incluyendo a la academia y a los medios de comunicación ^5^. Enfoques tradicionales de vigilancia, como el registro del número de casos y muertes, son insuficientes para las próximas amenazas sanitarias. Otro elemento clave será la cooperación entre los países, en especial entre los sudamericanos, para juntos afrontar de mejor manera y con enfoques modernos consensuados los retos venideros, adaptándonos a los cambios y reevaluando nuestras políticas en tiempo real, que es lo que demanda el control de las enfermedades infecciosas actuales.

Desde el INS, se dispone del Decreto Ley 1504 (aprobado el 2020) que tiene como fines «El fortalecimiento del Sistema Nacional de Salud para asegurar la articulación intra e intersectorial, intergubernamental, de las entidades públicas y privadas, y de la población para el cumplimiento de las políticas de salud; el fortalecimiento del sector salud, mediante la organización y gestión eficiente del Instituto Nacional de Salud a favor de la salud pública en el país; el mejorar la vigilancia y prevención de enfermedades, brotes, endemias, epidemias y pandemias en el territorio nacional; contribuir con el control de las enfermedades que afectan la vida de la población, así como mejorar la investigación, desarrollo e innovación, y las tecnologías en salud con la participación de instituciones públicas, privadas y el sector académico».

Con ese propósito, y entre otras iniciativas, actualmente se encuentra en marcha el proyecto de inversión para el «Mejoramiento y Ampliación de los Servicios brindados por el Sistema Nacional de Vigilancia en Salud Publica», cofinanciado por el Banco Mundial, que tiene como finalidad concretar el fortalecimiento de la capacidad resolutiva nacional frente a las enfermedades emergentes y reemergentes que vulneren la salud de la población, fortaleciendo tanto el INS, como a las 24 regiones y tres centros macrorregionales, en el Norte, Sur y Oriente, que podrán lograr una descentralización efectiva de los servicios especializados que brinda la institución.

Por último, considerando que en la década del 70 el INS contribuyó con la erradicación de viruela en el Perú y en otros países sudamericanos mediante la producción de la vacuna antivariólica liofilizada, se ha propuesto y apoyado iniciativas legislativas para contar con una planta de producción de vacunas que cuenten con plataformas tecnológicas modernas, y así dotar al país de capacidades técnicas para una respuesta oportuna para enfrentar, además de las pandemias, epidemias y endemias de enfermedades desatendidas. «Que la próxima pandemia nos encuentre mejor preparados como peruanos y como seres humanos».
